# Retraction Note: Numerical Simulation of Gravity Flow in Sublevel Caving Based on Polyhedron DEM

**DOI:** 10.1007/s42461-025-01420-z

**Published:** 2025-11-22

**Authors:** Changping Yi, Daniel Johansson, Matthias Wimmer, Anders Nordqvist, Jenny Greberg, Carlota Rodriguez San Miguel

**Affiliations:** 1 Division of Mining and Geotechnical Engineering, Luleå University of Technology, Luleå, Sweden; 2 Division of Mining and Geotechnical Engineering, Luleå University of Technology, Luleå, Sweden; 3 LKAB, Kiruna, Sweden

**Retraction Note: Mining**,** Metallurgy & Exploration (2023) 41:91–98**

10.1007/s42461-023-00903-1

The Editor-in-Chief has retracted this article at the request of the corresponding author because three of the authors listed, Matthias Wimmer, Anders Nordqvist and Jenny Greberg, did not contribute to this study. All authors agree with this retraction.

